# Role of Frontostriatal Connectivity in Adolescents With Excessive Smartphone Use

**DOI:** 10.3389/fpsyt.2018.00437

**Published:** 2018-09-12

**Authors:** Ji-Won Chun, Jihye Choi, Hyun Cho, Mi-Ran Choi, Kook-Jin Ahn, Jung-Seok Choi, Dai-Jin Kim

**Affiliations:** ^1^Department of Psychiatry, Seoul St. Mary's Hospital, The Catholic University of Korea College of Medicine, Seoul, South Korea; ^2^Department of Psychology, Korea University, Seoul, South Korea; ^3^Department of Radiology, Seoul St. Mary's Hospital, The Catholic University of Korea College of Medicine, Seoul, South Korea; ^4^Department of Psychiatry, SMG-SNU Boramae Medical Center, Seoul, South Korea

**Keywords:** excessive smartphone use, frontostriatal connectivity, cortisol, problematic internet use, withdrawal

## Abstract

As smartphone use has grown rapidly over recent decade, it has been a growing interest in the potential negative impact of excessive smartphone use. In this study, we aim to identify altered brain connectivity associated with excessive smartphone use, and to investigate correlations between withdrawal symptoms, cortisol concentrations, and frontostriatal connectivity. We focused on investigating functional connectivity in frontostriatal regions, including the orbitofrontal cortex (OFC), midcingulate cortex (MCC), and nucleus accumbens (NAcc), which is related to reward processing and cognitive control. We analyzed data from 38 adolescents with excessive smartphone use (SP) and 42 healthy controls (HC). In the SP group compared with HC, we observed lower functional connectivity between the right OFC and NAcc, and between the left OFC and MCC. Moreover, functional connectivity between the MCC and NAcc was greater in SP compared with HC. Subsequently, we examined the relationship between Internet use withdrawal symptoms, cortisol concentrations, and functional connectivity between the OFC and NAcc in SP and HC. We observed that more severe withdrawal symptoms were associated with higher cortisol concentrations in adolescents with excessive smartphone use. The most interesting finding was that we observed a negative correlation between OFC connectivity with the NAcc and both withdrawal symptoms and cortisol concentrations. The functional connectivity between the OFC and NAcc, and between the OFC and MCC are related to cognitive control of emotional stimuli including reward. The current study suggests that adolescents with SP had reduced functional connectivity in these regions related to cognitive control. Furthermore, Internet use withdrawal symptoms appear to elicit cortisol secretion, and this psychophysiological change may affect frontostriatal connectivity. Our findings provide important clues to understanding the effects of excessive use of smartphones on brain functional connectivity in adolescence.

## Introduction

Following recent developments in mobile communication technology, smartphones have become a necessity of everyday life beyond simple interpersonal communication. We use smartphone for various activities, such as information searching, online gaming, and social networking. Despite many benefits resulting from these developments, it has been reported that excessive mobile phone use could induce potentially risky behaviors, such as uncontrolled use and disturbance of adaptive behavior, which can have a negative impact on various aspects of daily life (1).

Nowadays, all kinds of media content are continuously available via portable mobile devices such as smartphones (2). It is possible that emotional sensitivity and protracted development of cognitive control during adolescence may make those at this stage of life more reactive to emotion-arousing media (3). A previous study reported that adolescents tend to use smartphones more often for Internet use than do adults, and they are more likely to be exposed to problematic smartphone use (4). Recently, cognitive neuroscience studies have used structural and functional magnetic resonance imaging (fMRI) to examine how the adolescent brain changes over course of adolescence (3). Given that brain regions involved in many social and cognitive functions are undergoing such broad changes during adolescence, it might be supposed that adolescents are greatly influenced by social interaction that occurs via the Internet.

It has been reported that negative aspects of excessive smartphone use are similar to Internet addiction, including Internet gaming disorder (5). In the previous study, Internet addiction and pathological Internet use have been revealed to induce negative outcomes such as uncontrolled Internet use, tolerance, withdrawal, social isolation, and poor academic or professional achievement (6). Previous research has suggested that individuals Internet addiction disorder (IAD) showed excessive use, tolerance, and withdrawal symptoms similar to substance use disorder (7). A study of the diagnostic criteria has revealed that 96% of individuals with IAD reported withdrawal symptoms (8). Moreover, a factor analysis of Internet addiction suggested that withdrawal symptoms are highest in Korean adolescents aged between 10 and 19 years (9). Withdrawal symptoms include anxiety about situations in which the Internet is not available and craving Internet use (9). In the previous case study of mobile phone dependence (MPD), individual with MPD might feel uncomfortable and annoyed in the absence of their mobile phone, including feeling a physical and psychological emptiness associated with withdrawal (10). Altogether, withdrawal symptoms are important factor in the pathological use of Internet. As a portable media for Internet use, the excessive use of the smartphone may be closely associated with the symptoms of the IAD.

To explore whether brain functional connectivity in the PFC is associated with withdrawal and cortisol concentrations might be helpful in understanding dysfunctional behavior associated with excessive smartphone use. Several previous studies have shown that anxiety experienced during real life stressful situations, as well as that induced by experimental situations, is related to increased cortisol levels (11–13). In previous studies of substance addiction, cortisol levels were positively correlated with withdrawal symptoms (13, 14). It is known that the hippocampus, amygdala, and prefrontal cortex (PFC) were associated with cortisol regulation in response to stress (15). The previous human studies related to the role of the PFC in cortisol regulation could find in functional neuroimaging studies investigating neural correlates of psychological stress processing (16–18). Previous neuroimaging studies of addictive behaviors have suggested a crucial role for the PFC in regulation of limbic regions and engagement of executive function, such as self-control, salience attribution, and awareness (19). In chronic drug use, it has been reported that corticolimbic areas such as the OFC and dorsal ACC located in midcingulate cortex (MCC) mediate processing of reward salience, motivation, and inhibitory control (20, 21). Previous studies have identified the OFC and ventral striatum (VS), including the nucleus accumbens (NAcc), as a set of reward-related brain structures (22), Another imaging study reported that compared with healthy controls, chronic cocaine abusers had lower metabolism in the right OFC and NAcc, which was related to cognitive inhibition (23). Previous structural neuroimaging studies have suggested the potential role of the NAcc in the excessive smartphone use. A diffusion tensor imaging (DTI) study has recently reported that individuals with smartphone dependence showed deficits in white matter structure such as internal capsule around NAcc, which was correlated with the severity of smartphone dependence (24). Moreover, another study revealed that high frequency of checking Facebook on the smartphone was associated with smaller gray matter volumes of the NAcc (25). Given the role of the OFC, MCC, and NAcc in reward processing and cognitive control, investigations of the functional connectivity among these regions has become key to understanding addictive behavior in excessive smartphone use.

Intrinsic functional connectivity acquired in resting-state fMRI can be defined as the temporal correlation of a neurophysiological marker measured in spatially different brain areas (26). Here, we focus on altered brain connectivity related to reward processing and cognitive control in the resting state in adolescents with excessive smartphone use compared with healthy controls. Additionally, we investigated correlations of functional connectivity of frontostriatal regions with Internet use withdrawal symptoms and increased cortisol concentrations.

## Methods

### Participants

In this study, we enrolled adolescent boys and girls aged 12–18 years by using online recruiting. A total of 801 adolescents responded to the online survey of smartphone use, and 127 adolescents and their parents expressed willingness to participate in the fMRI study. Subsequently, participants were divided into two groups (adolescents with excessive smartphone use, SP, and healthy controls, HC) according to an assessment by a clinician on the basis of the Korean Smartphone Addiction Proneness Scale (SAPS) (27) for Youth (for full details see section Clinical Assessments). Lastly, 80 adolescents passed MRI safety screening questionnaire.

The purpose and procedures of the study were explained to the participants and their parents prior to participation. Exclusion criteria involved past or current major medical disorders (e.g., diabetes mellitus), neurological disorders (e.g., seizure disorders, head injury) or psychiatric disorders (e.g., major mood disorders). All participants had normal or corrected-to-normal vision and were right-handed as evaluated by the Edinburgh Handedness Inventory (28).

For the fMRI study, 40 adolescents with SP (32 male and 8 female) and 40 HC (32 male and 8 female) were included. In order to screen out adolescents with current psychiatric diagnoses, all participants received the structured interview with the Korean Kiddie-Schedule for Affective Disorders and Schizophrenia (K-SADS-PL) (29) through a clinician. Of the adolescents with SP, one participant was excluded because of depressive disorder. Moreover, data from three participants were excluded because of severe head motion during acquisition. Therefore, data from 38 adolescents with SP (32 male and 6 females, mean age: 14.90 ± 1.49 years) and 38 HC (30 male and 8 females, mean age: 14.12 ± 1.34 years) were included in the analysis (Table 1). Each participant provided written informed consent in accordance with the Declaration of Helsinki, and the study protocol was approved by the institutional review board of Seoul St. Mary's Hospital. All experiments were performed in accordance with relevant guidelines and regulations.

**Table 1:** Demographic characteristics of the SP and HC.

	**SP (*****n*** = **38)**		**NC (*****n*** = **38)**		***t*-score**
	**Mean**	**SD**	**Mean**	**SD**	
Age	14.95	1.45	14.08	1.28	2.77^*^
K- WISC: block design	8.87	2.36	10.84	2.50	−3.54^*^
K- WISC: vocabulary	10.32	2.90	11.18	2.98	−0.20
Gender					
Male	84.2% (*n* = 32)		78.9% (*n* = 30)		*x*2 = 0.35
Female	15.8% (*n* = 6)		21.1% (*n* = 8)		
Time for smartphone using per week(h)	32.55	28.83	15.18	8.70	3.56^**^
SAPS	36.71	8.27	22.08	3.15	10.19^**^
Disturbance of adaptive function	13.42	3.09	8.13	1.65	9.31^**^
Withdrawal	8.45	3.78	5.32	1.40	4.79^**^
Tolerance	11.55	2.69	6.45	1.45	10.31^**^
K-scale	31.89	7.48	22.24	3.74	7.12^**^
Disturbance of adaptive function	9.82	3.19	7.05	1.77	4.67^**^
Withdrawal	8.87	2.45	6.37	1.85	4.96^**^
Tolerance	9.32	3.11	6.37	1.85	5.02^**^
BDI	12.26	9.61	6.34	4.80	3.40^*^
BAI	8.03	9.69	4.82	7.35	1.63

*SP, excessive smartphone use group; HC, Healthy control group; SAPS, Smartphone Addiction Proneness Scale; K-scale, Korean Internet Addiction Proneness Scale; BDI, Beck's Depression Inventory; BAI, Beck's Anxiety Inventory*.

* *p < 0.01*,

** *p < 0.001*.

### Clinical assessments

Excessive smartphone use was estimated with the Korean SAPS for Youth (27). Investigation of the reliability of the scale yielded a Cronbach's alpha of 0.88. The SAPS is a self-report scale that includes 15 items, and responses are scored on a four-point Likert scale (1: Not at all to 4: Always). The SAPS has four subscales: disturbance of adaptive functions, virtual life orientation, withdrawal, and tolerance. Participants were classified as SP if their total score exceeded 42, or if their subscale scores exceeded 14, 12, and 13 for disturbance of adaptive function, withdrawal, and tolerance, respectively. Otherwise, participants were classified as HC.

Additionally, severity of problematic Internet use was estimated with the Korean Internet Addiction Proneness Scale (the K-scale) developed by the South Korean government in 2002 (30). The K-scale includes 15 items and responses are scored on a four-point Likert scale (1: Not at all to 4: Always). The K-scale has seven subscales: daily life disturbance, disturbance of reality testing, automatic addictive thoughts, virtual interpersonal relationships, deviant behavior, and tolerance. The reliability and validity of the K-scale have been established for elementary school, and middle and high school students (31).

Finally, severity of depressive symptoms was assessed with the Beck's Depression Inventory (32) and severity of anxiety symptoms was assessed with the Beck's Anxiety Inventory (33). A brief assessment of Intellectual functioning was conducted using the Vocabulary and Block Design subtests of the Korean-Wechsler Intelligence Scale for Children, 4th edition (K-WISC- IV) (34). These two subtests have good reliability and high correlation with the full-scale scores of WISC (35). All participants completed the vocabulary and block design which were the subtests for the verbal comprehension index and perceptual reasoning index, respectively.

### Physiological assessments

Blood samples from all participants were collected in the afternoon (between 13:00 and 15:00) and kept at room temperature for 2 h before being centrifuged at 1,000x g for 15 min. The upper phase (serum) was transferred into a fresh tube. Serum was stored at −80°C until immunoassay was performed. Cortisol levels were analyzed with the Human Circadian/Stress Magnetic Bead Panel (HNCSMAG-35 K, EMD Millipore, Billerica, MA, USA) according to the manufacturer's instructions. In brief, 25 μL antibody-immobilized beads were added to each well containing standard and serum samples, and the plate was incubated overnight at 4°C. After washing with 200 μL wash buffer, 50 μL detection antibody was added to each well and the plate was incubated at room temperature for 1 h. Fifty microliters streptavidin-phycoerythrin was added to each well and the plate was incubated at room temperature for 30 min. After washing with 200 μL wash buffer, 150 μL sheath fluid was added to each well and the plate was read on the Luminex 200TM (EMD Millipore, Billerica, MA, USA).

### MRI data acquisition

Functional and structural MRI data were acquired with a 3-Tesla MAGNETOM Verio system (Siemens, Erlangen, Germany) equipped with a 16-channel head coil. Participants' heads were cushioned with attached earmuffs. Functional images were obtained with a T2^*^-weighted gradient echo echo-planar imaging sequence: repetition time (TR) = 2,000 ms, echo time (TE) = 30 ms, voxel size = 3.59 × 3.59 × 3.60 mm, matrix size = 64 × 64, and slice number = 31. During scanning, participants were instructed to fixate their eyes on a crosshair and to remain as motionless as possible at rest. Structural images with a resolution of 1 × 1 × 1 mm were acquired with a 3D T1-weighted gradient echo sequence (176 slices, TR = 1,780 ms, TE = 2.22 ms, and image matrix = 256 × 256).

### Functional connectivity analysis

Resting-state fMRI data were preprocessed with SPM12 (http://www.fil.ion.ucl.ac.uk/spm/). Functional images were corrected for slice-timing and head motion, and spatially normalized to the same coordinate frame as the Montreal Neurological Institute template brain. They were subsequently, spatially smoothed with a Gaussian kernel of 6 mm full width at half maximum. A nonlinear deformation field for spatial normalization was derived from the segmentation of the structural MRI volume coregistered to the mean of the realigned resting state fMRI volumes. Additionally, nuisance covariates, including six head movement parameters estimated during realignment of the functional images.

Region of interest (ROI)-to-ROI functional connectivity network analyses were performed with in-house software (the Intuitive Resting-state Functional Connectivity toolbox, iRSFC, https://github.com/skyeong/iRSFC) running on MATLAB R2011b (The MathWorks Inc., Natick, MA). First, linear trends of the time courses were removed and temporally band-pass filtered (0.009–0.08 Hz) to denoise the signals, removing physiological noise and low frequency signal drifts. Nuisance covariates, including six head movement parameters estimated during realignment of the functional images, as well as global, cerebrospinal fluid, and white matter signals were regressed out.

**Figure 1:**
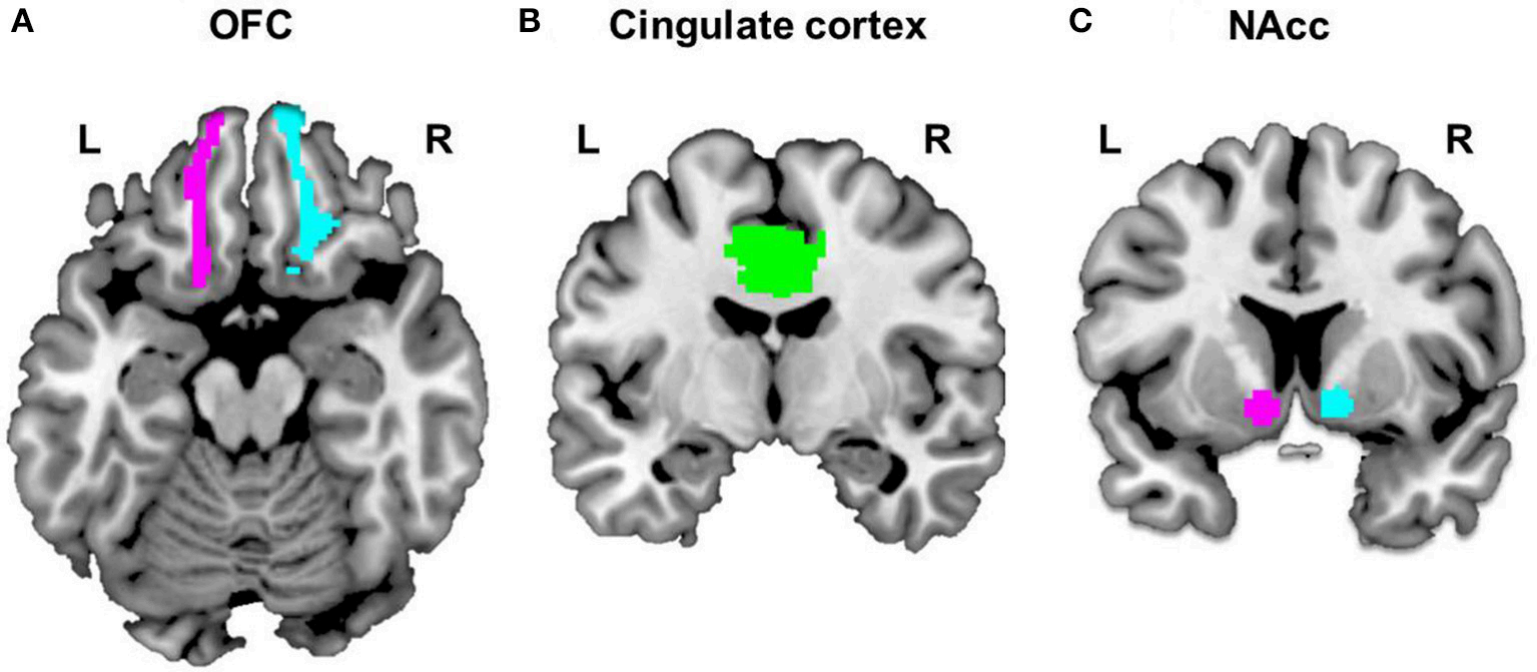
Selected ROIs. Five regions of Interest (ROIs) were selected to construct each subject's ROI-to-ROI as follows: the bilateral OFC **(A)** and cingulate cortex **(B)**, extracted from the automated anatomical labeling (AAL) brain atlas and bilateral NAcc **(C)**, extracted from the probabilistic Harvard-Oxford subcortical atlas (thresholded at 50%).

To construct each participant's ROI-to-ROI functional connectivity networks, we selected five ROIs as follows: the left OFC, right OFC, and midcingulate cortex (MCC) (Figure 1), extracted from the automated anatomical labeling (AAL) brain atlas (36), and the left and right NAcc (Figure 1C), extracted from the probabilistic Harvard-Oxford subcortical atlas (thresholded at 50%). We focused on the OFC because of its role in cognitive regulation (37) and decision making (38). Moreover, it has been reported that the MCC in AAL brain atlas, a key region comprising the salience network (39), is implicated in conflict monitoring (40). We generated specifically the MCC ROI, based on the AAL brain atlas, which is combined the left and right hemispheres because the MCC is located to medial section in the brain. Given reward and stimuli sensitivity observed in excessive smartphone users, we focused on the role of the NAcc related to reward processing (41, 42) or reward expectation (43). We calculated correlation coefficients between the time series of these five regions, which were then transformed to z-values by using Fisher r-to-z transformation. The outputs of the ROI-to-ROI functional connectivity network analyses represent the matrix of the connection strength between the five ROIs.

A two-sample *t*-test was conducted on each participant's functional connectivity network for group comparisons. We analyzed functional connectivity between ROIs within hemispheres because of probability of functional lateralization that have been reported in previous studies (44, 45). The significance level was determined at a *p*-value of 0.05 with the false discovery rate procedure for correcting multiple comparisons over the ROIs in each hemisphere. Furthermore, we examined the relationship between Internet use withdrawal, cortisol levels, and functional connectivity strength in each group. Results of the correlation analyses were transformed to z scores using Fisher's r-to-z for group comparisons.

## Results

### Clinical and physiological data

Table 1 present the demographic and clinical characteristics of the two groups. The two groups did not show significant differences in scores for the vocabulary subtest of the K-WISC, the distribution of gender, the BAI scores and cortisol concentrations. Compared to HC, the SP was significantly greater in age [*t*_(74)_ = 2.77, *p* < 0.01], time spent using a smartphone per week [*t*_(74)_ = 3.56, *p* < 0.005], SAPS scores [*t*_(74)_ = 10.19, *p* < 0.001], K-Scale scores [*t*_(74)_ = 7.12, *p* < 0.001], and BDI scores [*t*_(74)_ = 3.40, *p* < 0.005] and lower in scores for block design subtest of the K-WISC [*t*_(74)_ = −3.54, *p* < 0.005]. In particular, adolescents with SP, compared to HC, had greater scores for disturbance of adaptive functions [*t*_(74)_ = 9.31, *p* < 0.001], withdrawal [*t*_(74)_ = 4.79, *p* < 0.001], and tolerance [*t*_(74)_ = 10.31, *p* < 0.001] subscales of the SAPS. Similarly, adolescents with SP, compared to HC, had greater scores for disturbance of adaptive functions [*t*_(74)_ = 4.67, *p* < 0.001], withdrawal [*t*_(74)_ = 4.96, *p* < 0.001], and tolerance [*t*_(74)_ = 5.02, *p* < 0.001] subscales of the K-Scale.

### Functional connectivity

**Figure 2:**
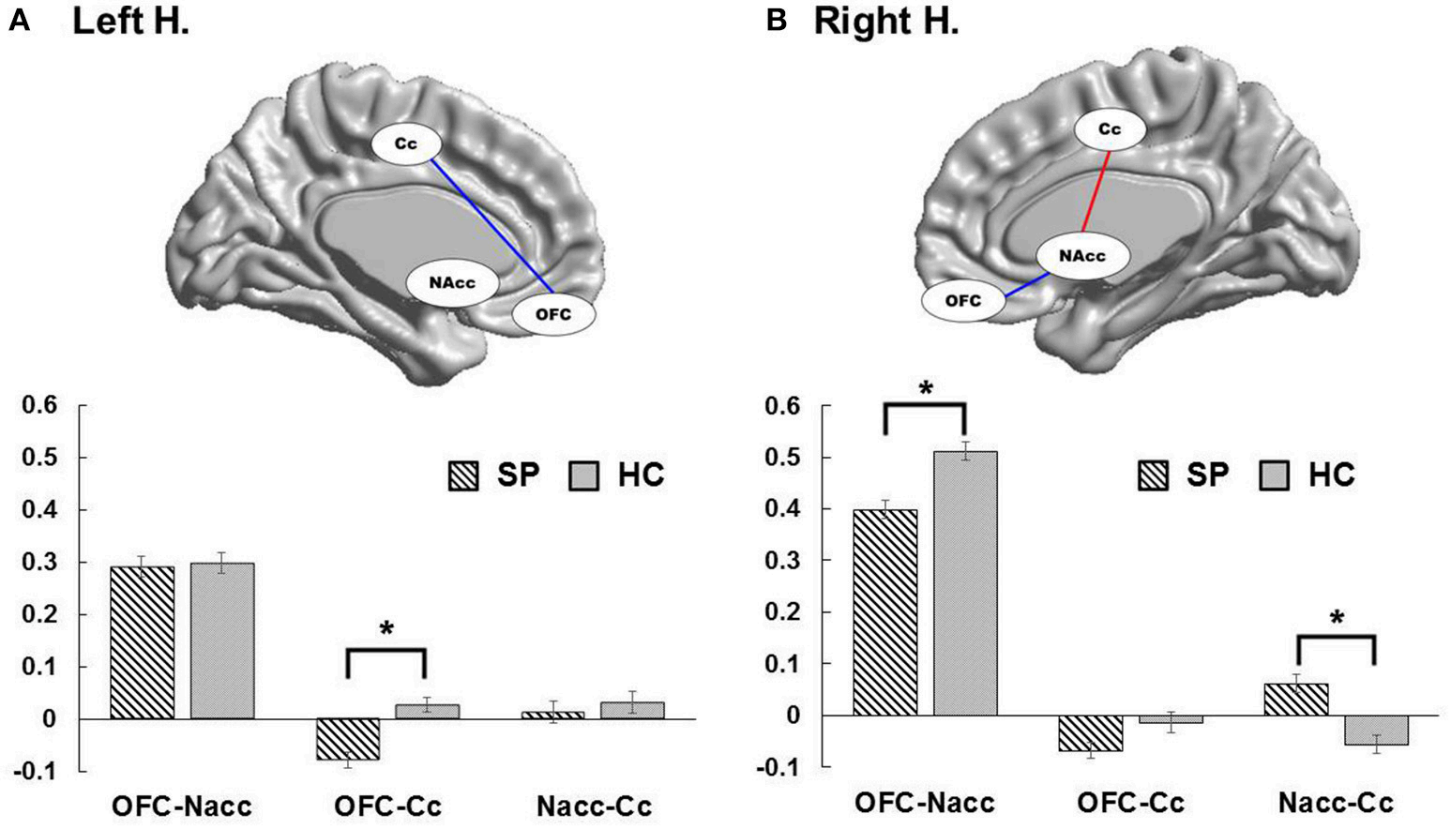
Group differences of functional connectivity in each hemisphere. In the left hemisphere, adolescents with SP had decreased connectivity between the OFC and cingulate cortex compared with HC **(A)**. In the right hemisphere, adolescents with SP showed weaker OFC connectivity with the NAcc in compared with HC, whereas they revealed stronger connectivity between the NAcc and cingulate cortex in compared with HC **(B)**.

Figure 2 present group differences in ROI-to-ROI functional connectivity. To explore brain imaging markers underlying excessive smartphone use, we examined functional connectivity between the left and right OFC, MCC, and left and right NAcc in each hemisphere in adolescents with SP and HC. In the right hemisphere, we observed weaker the right OFC connectivity with the right NAcc in adolescents with SP compared with HC [*t*_(74)_ = −2.25, corrected *p* < 0.05], whereas there was stronger connectivity between the right NAcc and MCC in adolescents with SP compared with HC [*t*_(74)_ = 2.42, corrected *p* < 0.05]. There was no significant difference in the right OFC and MCC connectivity between the two groups. In the left hemisphere, adolescents with SP had lower connectivity between the left OFC and MCC compared with HC [*t*_(74)_ = −2.47, corrected *p* < 0.05]. There were no other significant differences observed in the left hemisphere.

To consider the effect of potential confounds in group comparisons, we performed ANCOVA with age, the scores of the block design and BDI as covariate of no interest to adjust for the effects of the clinical variables when comparing the functional connectivity between the two groups. The functional connectivity between the right OFC and right NAcc [*F*_(1, 73)_ = 5.79, *p* < 0.05], the right NAcc and MCC [*F*_(1, 73)_ = 5.61, *p* < 0.05], and the left OFC and MCC [*F*_(1, 73)_ = 4.87, *p* < 0.05] were still significantly different between the two groups, after adjusting for age. When adjusting for the BDI scores, between-group differences in functional connectivity between the right NAcc and MCC [*F*_(1, 73)_ = 4.93, *p* < 0.05], and the left OFC and MCC [*F*_(1, 73)_ = 5.47, *p* < 0.05] remained significant, but the right OFC-NAcc functional connectivity showed a trend toward significance [*F*_(1, 73)_ = 3.49, *p* = 0.066]. Lastly, after adjusting for the scores of the block design, between-group differences in functional connectivity between the right OFC and NAcc [*F*_(1, 73)_ = 4.17, *p* < 0.05] and the right NAcc and MCC [*F*_(1, 73)_ = 5.42, *p* < 0.05] presented significance, whereas the left OFC-MCC functional connectivity was not significantly different between the groups [*F*_(1, 73)_ = 2.39, *p* = 0.126].

### Correlations

**Figure 3:**
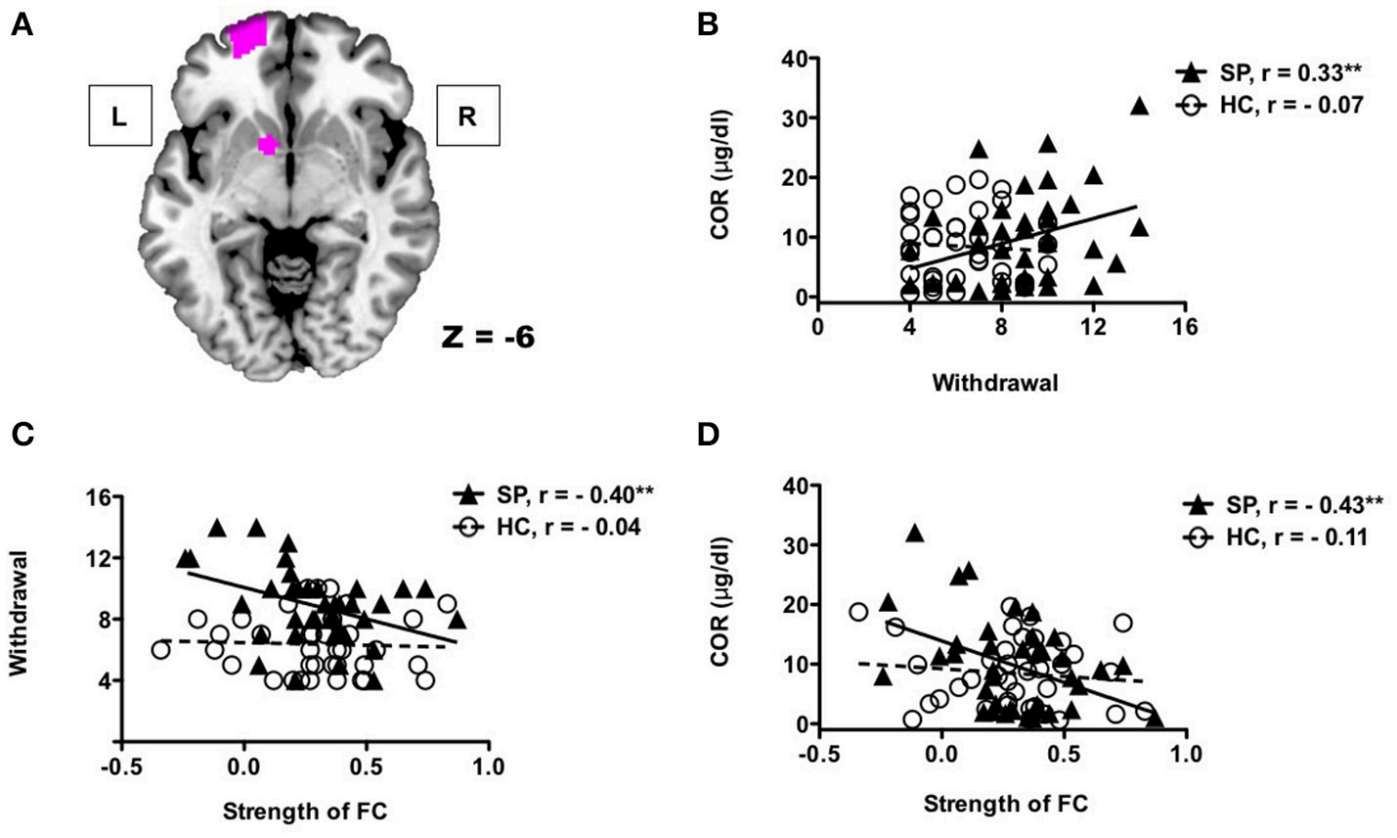
Correlations between withdrawal symptoms, cortisol concentrations, and functional connectivity The left OFC and NAcc **(A)**. Adolescents with SP showed positive correlation between Internet use withdrawal symptoms and cortisol concentrations **(B)**. There were significant correlations between frontostriatal connectivity of the left hemisphere and Internet use withdrawal symptoms **(C)**, and between frontostriatal connectivity and cortisol concentrations **(D)** in adolescents with SP.

Figure 3 presents the results of the correlation analyses between withdrawal symptoms, cortisol concentrations, and the left frontostriatal connectivity. The relationship between Internet use withdrawal symptoms and cortisol concentrations was significantly correlated in adolescents with SP (*r* = 0.33, *p* < 0.05), but not in HC did not (*r* = −0.07, *p* = 0.68), and the correlation coefficients were statistically different between the two groups (*z* = 1.73, *p* < 0.05). Internet use withdrawal symptoms was negatively correlated with the left frontostriatal connectivity in the adolescents with SP (*r* = −0.40, *p* < 0.05), but not in HC (*r* = −0.04, *p* = 0.80), whose correlation coefficients was significantly different between the groups (*z* = 1.6, *p* < 0.05). Lastly, the left frontostriatal connectivity was negatively correlated with cortisol concentrations in adolescents with SP (*r* = −0.43, *p* < 0.01), not in HC (*r* = −0.18, *p* = 0.48). However, these correlation coefficients were not significantly different between the groups (*z* = −1.43, *p* = 0.07).

## Discussion

In recent years, the use of smartphones has increased rapidly, and the negative phenomenon of excessive use of smartphones, including dependency, problematic use, and addictive behaviors (46), have been reported. Excessive Internet and smartphone use has become problematic for adolescents who may experience negative emotional, cognitive, and physical states during and after use (46). Resting-state connectivity analysis allows us to identify altered intrinsic functional connectivity in brain regions associated with cognitive control and affective-motivational processes in adolescents with excessive smartphone use (47). In this study, we aimed to identify altered brain connectivity associated with excessive smartphone use, and investigated correlations among withdrawal symptoms, cortisol concentrations, and frontostriatal connectivity.

The results of this study indicated that adolescents with SP had lower functional connectivity between the right OFC and NAcc, and between the left OFC and MCC. It is known that brain development in adolescence is associated with gradual improvements in cognitive control, which is related to PFC involvement. However, there is also heightened reward responsiveness to social and affective stimuli related to increased activity in the VS (47). Previous studies have reported that the NAcc is linked to reward anticipation and the OFC is related to decision making in reward processing (48–50). These previous findings highlight that the NAcc may be responsible for affective signals of reward and use these to modulate learning of reward associations (51, 52). In contrast, the OFC mostly monitors and evaluates reward outcomes (51, 52). Given the role of the PFC and VS in reward processing, weakened connectivity between the PFC, including the OFC, and the VS might cause impaired top-down executive control of impulsiveness. Moreover, in the previous study using DTI, SP had significantly lower white matter integrity in internal capsule compared to HC, which was associated with the severity of smartphone dependence (24). These previous results are consistent with our findings related to functional abnormality in NAcc.

In a previous structural imaging study, participants with internet gaming disorder had reduced gray matter volumes in the ACC and orbitofrontal PFC, suggesting that internet gaming disorder is related to both functional and structural alterations in frontocingulate regions (53). Moreover, previous research has suggested that cocaine abusers have reduced OFC responsivity when controlling drug-taking behavior (54), and research using positron emission tomography identified decreased metabolism in the OFC induced by inhibition cues of craving (23). In previous studies of substance addiction, reduced regional activity in the OFC and cingulate gyrus were associated with decreased dopamine function (55). Previous findings also suggest that dopamine responses in individuals with substance abuse induced functional impairment of the OFC and ACC similar to those in patients with depression (56). Moreover, in a study using emotional faces, individuals with SP showed lower activity in frontocingulate regions compared with healthy individuals (57). Therefore, it could be supposed that intrinsic functional connectivity between the OFC and NAcc, and between the OFC and MCC are connected with cognitive control of emotional stimuli including reward. Our results indicated that adolescents with excessive smartphone use revealed lower functional connectivity in regions related to cognitive control compared to HC.

In the results of the group comparisons including covariates, we did not observe the functional connectivity between left OFC and MCC following adjustment for the scores of the block design. Block design is designed to assess problem solving, space perception, and visual processing. This finding, thus, could explain that intelligence domain related to perceptual reasoning might be associated with the frontocingulate connectivity implicated in cognitive control. In the further work, it would be important to investigate the effect of smartphone dependency on the association between perceptual reasoning and prefrontal functional connectivity. We also observed the marginal effect of the BDI on the group difference in the right frontostriatal connectivity. Given the previous results reporting the relationship between depression and IA (58, 59), the functional connectivity study related to depression in adolescents with smartphone dependency would seem to be worth.

In this study, functional connectivity between the MCC and NAcc was greater in adolescents with SP compared with HC. The role of dorsal ACC located in MCC includes monitoring for cognitive control (40) and guiding reward-based decision making (60). Moreover, the MCC, which is related to salience of stimuli, regulates responses by providing updated predictions of expected cognitive demands (61). In terms of functional connectivity with the NAcc, it could suggest that the MCC plays a role in monitoring signals related to reward. Therefore, greater functional connectivity between the MCC and NAcc in adolescents with SP compared with HC may reflect heightened monitoring based on reward processing in the resting state.

Adolescents are more likely to exhibit problematic smartphone use patterns after substituting a smartphone for the Internet (62). In this study, we identified that higher withdrawal symptoms were related to higher cortisol concentrations in adolescents with SP compared to HC. It was reported that withdrawal induced by drug involve the emergence of negative emotion state, characterized by an inability to experience pleasure from common non-drug related rewards. (56). Previous research suggests that the central pathology underlying IAD might be more similar to addiction than a disorder of impulse control (7). The Internet use withdrawal symptoms were correlated with both left frontostriatal connectivity and cortisol concentrations in adolescents with SP compared to HC. Cortisol plays a key role in the physical adaptation to increased energy demands during stress period (15). In a previous study, patients undergoing alcohol withdrawal showed increased cortisol concentrations (63). It was known that the OFC collect and integrate sensory information from the body and the environment, and participates in controlling one's emotional state (64). In the previous study, decreased OFC activity is connected with increased cortisol level in response to a stress task (65). It was reported that cortisol induced coordinated stress response in the PFC (66). Given the role of the OFC in cortisol secretion (67, 68), Internet use withdrawal symptom will likely lead to cortisol secretion, and this psychophysiological change might subsequently affect frontostriatal connectivity. In the frontostriatal connectivity, we focused on the role of NAcc in reward processing. Previous research using reward paradigms have reported enhanced neural activity in the VS of adolescents in response to monetary rewards (69), and it was suggested that this activity related to heightened sensitivity to social reward (3). Social reward sensitivity might be a strong motivation for social media use and could instigate Internet use via a smartphone in adolescents. Therefore, it could explain that the negative correlation between the frontostriatal connectivity and withdrawal symptoms in SP is related to cognitive deficits of reward responsiveness that accompany withdrawal from smartphone dependence.

A smartphone has included various applications that require Internet access (24). Thus, excessive smartphone use could cause physical, mental and psychosocial problems similar to Internet addiction (70). On the other hands, it has been reported that the specific sources of addictive content were difference between excessive smartphone use and Internet addiction (71). In this study, we investigated excessive smartphone use including Internet use. Smartphone is almost portable, have quick access to information, and communicate instantly. Therefore, it can be inferred that the results of this study reflect factors related to immediate and sustained response compared to widespread Internet addiction.

Finally, several important limitations need to be considered. First, although we controlled for comorbidities such as attention deficit hyperactivity, depression, and anxiety through clinical interviews, various psychological and environmental variables of participants were not considered. Second, the main contents of the smartphone use were not considered in this study. A future study with a stronger focus on the effect of the specific content of smartphones, such as games or social network service, is therefore required. Lastly, we have to consider further work associated with a longitudinal study of the brain development in adolescents in other to validate the cause and effect of excessive smartphone use.

In summary, we used a functional connectivity analysis to identify regional connectivity related to cognitive control and reward prediction in adolescents with SP, and investigated functional connectivity in such adolescents compared with HC. In adolescents with SP, functional connectivity between the OFC and NAcc, and between the OFC and MCC was lower compared to HC. Furthermore, we observed less functional connectivity between the OFC and NAcc related to withdrawal symptoms and cortisol secretion. Our findings suggest that excessive smartphone use is related to altered functional connectivity between regions related to cognitive control and reward prediction. The understanding the brain regions that show altered functional connectivity might be helpful for developing effective interventions to control Internet use in adolescents.

## Author contributions

D-JK and J-WC contributed to the conception and design of study. J-WC and JC contributed to the acquisition of imaging data. HC undertook the clinical assessments. J-WC and JC performed imaging data analysis. J-WC wrote the manuscript including the figures and tables. JC and M-RC assisted with the explanation of data and contributed to the final draft of the manuscript. HC, K-JA, J-SC, and D-JK contributed revising the manuscript logically for important theoretical content. All authors contributed to the manuscript and have approved the final manuscript.

### Conflict of interest statement

The authors declare that the research was conducted in the absence of any commercial or financial relationships that could be construed as a potential conflict of interest.
